# TIM-3^+^ T cells are not exhausted but activated cells in the tumor microenvironment

**DOI:** 10.1186/2051-1426-1-S1-P190

**Published:** 2013-11-07

**Authors:** Hyun-Bae Jie, Jing Li, Raghvendra Srivastava, Robert L Ferris

**Affiliations:** 1 Otolaryngology, University of Pittsburgh, Pittsburgh, PA, USA; 2 Immunology, University of Pittsburgh, Pittsburgh, PA, USA; 3 Cancer Immunology Program, University of Pittsburgh Cancer Institute, Pittsburgh, PA, USA

## 

Although T-cell immunoglobulin mucin 3 (TIM-3) does not contain inhibitory or death signaling motifs in its cytoplasmic domain, it has been proposed to be associated with T cell suppression and/or exhaustion. However, several lines of evidence suggest that TIM-3 can stimulate T cells as a costimulatory molecule by coupling Src family tyrosine kinase Fyn and the p85 phosphatidylinositol 3-kinase (PI3K) adaptor to TCR signaling. We examined the expression pattern and function of TIM-3 and other immune checkpoint receptors, CTLA-4 and PD-1 on tumor infiltrating lymphocytes (TIL), compared to those of peripheral blood T lymphocytes (PBL) in patients with head and neck cancer (HNC). Here, we report that TIM-3^+^CD8^+^ TIL express higher granzyme B/perforin, more actively proliferate under anti-CD3/-CD28 stimulatory conditions, and are more resistant to activation induced cell death than TIM-3^-^CD8^+^ TIL, indicating TIM-3 can positively regulate T cell responses. Analysis of downstream signaling molecules including phosphorylated JAK/STAT-1, PD-1/SHP-2, and costimulatory CD137 in CD8^+^ TIL subsets supports our observation that TIM-3^+^CD8^+^ TIL are activated cells in HNC patients. However, PD-1 and CTLA-4 can negatively regulate immune responses of TIM-3^+^CD8^+^ and TIM-3^+^CD4^+^ TIL respectively. More importantly, neoadjuvant immunotherapy of HNC patients with the EGFR-specific mAb cetuximab increased both TIM-3 and PD-1 expression on CD8^+^ TIL, which was correlated with higher granzyme B/perforin expression in TIM-3^+^CD8^+^ TIL. Taken together, these findings suggest that TIM-3 functions as a positive regulator of activated T cells in the tumor microenvironment while CTLA-4 and PD-1 modulate the function of activated TIM-3^+^ TIL. We therefore suggest that TIM-3 can be used as a biomarker to indicate activation status of T cells in the tumor microenvironment depending on PD-1 co-expression, particularly in response to cancer therapy including cetuximab-based immunotherapy.

